# “Desire Is Like a Dreadful Monster”: Analysis of Extended Metaphors in L2 Argumentative Essays by Chinese Learners of English

**DOI:** 10.3389/fpsyg.2021.803359

**Published:** 2021-12-20

**Authors:** Qiuyun Lu

**Affiliations:** ^1^School of Foreign Studies, Yangtze University, Jingzhou, China; ^2^School of Education, University of Leeds, Leeds, United Kingdom

**Keywords:** extended metaphors, systematic metaphors, L2 argumentative essays, metaphoric competence in L2, stimulated recall comments, communicative functions

## Abstract

This article explores the use, function, and understanding of extended metaphors in L2 argumentative essays by Chinese learners of English. The analysis starts with the identification of linguistic metaphors and extended metaphors in 72 argumentative texts produced by 37 intermediate Chinese English majors. The function of extended metaphors is then analyzed by adopting the bottom-up approach of establishing systematic metaphors from those identified extended metaphors, to draw learners’ communicative intentions in producing extended metaphors. To understand learners’ thinking processes behind using extended metaphors while writing, four of nine writers were interviewed about the process of writing extended metaphors in their texts in the stimulated recall interviews. It is found that extended metaphors, expressed through similes or direct metaphors at strategic stages in L2 argumentative essays, are often the result of learners’ conscious manipulation of L1 in producing L2 for various communicative purposes, such as the desire for vividness, coherence, comprehensibility, when there is a knowledge gap between L1 and L2, and for evaluative and persuasive power. These communicative functions are consistent with the ideational, interpersonal, and textual functions of language, which also coincide and interact with the rhetorical goals of moves and stages in L2 argumentative essays. Metaphoric thinking, L1 influence, and struggling to express meaning and persuade, cited in learners’ thought reports, are major factors triggering extended metaphors. The findings of this article can contribute to the knowledge of learners’ metaphoric competence in L2, which can, in turn, enrich teachers’ metaphor knowledge and draw teachers’ attention to learners’ creative ways of using metaphors and then raise metaphor awareness in L2 writing, teaching, and learning.

## Introduction

Metaphor is understood as a tool of describing or viewing something abstract, i.e., topic domain, in terms of something more concrete, i.e., vehicle domain, in the applied linguistic tradition (Cameron, 2003; Low et al., 2008; Deignan, 2017). Metaphor has been demonstrated to be pervasive in language generally, as well as in academic writing specifically (Lakoff and Johnson, 1980; Power and Carmichael, 2007; Herrmann, 2013; Littlemore et al., 2014; Hoang, 2015; Hoang and Boers, 2018; Nacey, 2020). Researchers find that metaphor can be described as opportunities of achieving more expressive or pervasive power in argumentative writing for L2 learners with different language backgrounds at different language levels, e.g., Spanish (MacArthur, 2010), Norwegian (Nacey, 2013), German and Greek (Littlemore et al., 2014), and Thai (Hoang, 2015; Hoang and Boers, 2018). For instance, MacArthur writes, “there are no ‘correct’ or ‘incorrect’ metaphors for among all the forces that drive semantic extension, the most powerful is metaphor…” (MacArthur, 2010, p. 159). Littlemore et al. (2014, p. 120) suggest that “one might expect development in the production of metaphor clusters in learners’ writing at the different levels.” An example of metaphor clustering in Littlemore et al.’s (2014) research is produced by an advanced German speaker of English (hereafter, linguistic metaphors are underlined):

*[…] your heath [health] will suffer when you reath [reach] a higher age. An old car doesn’t run as smooth as a new one. This will sooner or later reduce your quality of life.*

Littlemore et al. (2014, p. 136) argue that “the learner is able to use a creative direct metaphor for humorous effect, which makes their writing even more persuasive, by comparing an old person with an old car.” With the ethical approval granted by the ESSL, Environment and LUBS Faculty Research Ethics Committee, University of Leeds (AREA 16-160), creative metaphor uses were observed which were produced by an intermediate Chinese learner of English in their writing assignment:

*It’s our duty to purify our standard language and maintain our culture purity. We should not use Internet buzzwords without limit and make our language lose its own original appearance (Chen, a second-year Chinese university student studying in English language).*

In this example, an extended analogy between the Chinese language and human beings is summarized in a personification metaphor: *LANGUAGE IS A PERSON*. The learner draws multiple parallels between language and human beings, such as the metaphorically used words of “purity” and “appearance.” Creative direct metaphors or personification metaphors like these show learners’ ability to “express themselves and to create meaning in a second language by means of metaphor” (Postma, 2015, p. 49), or learners’ metaphoric competence in L2 (Birdsell, 2018).

The tendency for metaphors to extend or cluster at certain points in texts or discourse has been noted by some metaphor researchers (Corts and Pollio, 1999; Corts and Meyers, 2002; Cameron, 2003; Koller, 2003; Cameron and Stelma, 2004; Corts, 2006; Semino, 2008; Kathpalia and Carmel, 2011; Krennmayr, 2011; Littlemore et al., 2014; Dorst, 2017; Sun and Chen, 2018). The widespread and intriguing phenomenon where speakers or writers suddenly produce multiple metaphors in close proximity in texts or discourses has been defined as the pattern of metaphor clustering or metaphor clusters (Cameron and Stelma, 2004; Semino, 2008). Investigations on functions of metaphor clusters in spoken and written contexts, such as lectures (Corts and Pollio, 1999), sermons (Corts and Meyers, 2002), political speeches (Semino, 2008), news articles (Krennmayr, 2011), and business magazines (Koller, 2003), find that metaphor clusters occur at particularly significant points in texts or discourses and relate to a range of communicative functions. “Metaphor clusters are often used in strategic positions for rhetorical purposes” (Semino, 2008, p. 24). Kimmel summarizes three functions of metaphor clusters by reviewing prior studies on metaphor clusters in written texts: “(1) metaphor clusters are attention-grabbing and thus a relevance-producing device; (2) clusters seem to occur ‘where the action is;’ and (3) metaphor clusters connect and dynamize discourse” (Kimmel, 2010, p. 98).

This article focuses on one type of metaphor cluster—extended metaphors, from which systematic relationships among related vehicle terms of linguistic metaphors can be identified out (Semino, 2008; Maslen, 2017). The extension of linguistic metaphors often involves “a single metaphoric idea over a long stretch of language” (Denroche, 2018, p. 7), or systematic metaphors by establishing related vehicle terms (Cameron et al., 2010), such as the metaphoric idea *LANGUAGE IS A PERSON^1^*, established from the clustering of connected linguistic metaphors produced by Chen above. Discussions on the role of extended metaphors in texts and discourses have involved some metaphor scholars (e.g., Darian, 2000; Semino, 2008; Carter and Pitcher, 2010; Goatly, 2011; Naciscione, 2016; Thibodeau, 2017; Denroche, 2018). Semino (2008), Goatly (2011), and Thibodeau (2017) put the emphasis on the “text structuring” or organizing role of extended metaphors in texts and discuss the pervasive power of extended metaphors. Naciscione regards extended metaphors as a structure/pattern of figurative thought, which “helps to form new creative instantiations in use” (Naciscione, 2016, p. 243). Denroche (2018) also suggests that extended metaphors are likely to involve novel or creative metaphorical ideas. The link between metaphoric thinking, creativity, and extended metaphors has been discussed in these studies. In the context of language teaching and learning, Darian (2000) argues that the use of extended figurative language is a way of metaphoric thinking, which is productive for students to discuss ideas in writing; and the use of direct metaphors is helpful to students in understanding complex ideas in science, such as those easy-to-understand direct analogies, simile forms, personifications, and animations in science texts. For instance, students could “think of electricity as analogous to the flow of water” (Darian, 2000, p. 183). Carter and Pitcher (2010), inspired by the role of extended metaphors in the scaffolding learning in electronic subjects, explore how to use metaphors as a pedagogical aid in helping students in thesis writing, by finding similarities and differences between the vehicle domain and the target domain.

As noted above, prior researchers tend to choose topic-based (argumentative) writing texts to investigate metaphor production in L2 (Chapetón, 2010; MacArthur, 2010; Nacey, 2013, 2017; Littlemore et al., 2014; Hoang, 2015; Gao, 2016), and to compare metaphor uses in native and non-native writing (Chapetón-Castro and Verdaguer-Clavera, 2012). Possible reasons are: first, argumentative writing topics are often abstract and reflective, which can “involve a substantial amount of metaphor” (Littlemore et al., 2014, p. 121), and “some topics may also trigger more metaphor than others” (Nacey, 2020, p. 296); second, “metaphors have been important argumentative and rhetorical devices such as creating vivid images and function as examples or organizing ideas behind a series of examples” (Klebanov and Flor, 2013, p. 11). Research on metaphor and L2 topic-based writing has shown that learners have the need to use figurative language to express complex and abstract ideas and will do so to fulfill communicative needs in L2 argumentative writing, such as the persuasive argument constructed by the German English learner’s comparison between “an old person” and “an old car” (Littlemore et al., 2014), and the Chinese English learner’s metaphorical comparison between “a short-sighted person” and “a frog in the well” when arguing about the importance of being knowledgeable and broad-minded (Xu and Tian, 2012). Following the literature, it seems safe to conclude that as in many English as a Second Language (ESL) contexts, argumentative writing is crucial for Chinese university students to succeed in high-stakes examinations (Liu and Stapleton, 2014; Abdollahzadeh et al., 2017) and extended metaphors are productive in driving semantic extension and organizing ideas when students are under communication pressure (Darian, 2000; MacArthur, 2010).

This article explored the use of extended metaphors in L2 argumentative essays by Chinese university students and students’ thought reports behind some of their extended metaphor uses, given the fact that relatively little is known about the use and function of extended metaphors in non-native English learners’ argumentative writing; and about “whether or not a writer has deliberately used metaphor in this way or whether they have done so subconsciously” (Littlemore et al., 2014, p. 137). The term “extended metaphor” was used “when at least two metaphorically used words belonging to different phrases describe the same topic domain in terms of the same vehicle domain” (Semino, 2008, p. 25). Here, terms of “topic” and “vehicle” are used for basic descriptive reporting (Low et al., 2008; Maslen, 2017). Hyland’s (1990) model of describing the rhetorical structure of an ESL argumentative essay was adopted which divides an ESL argumentative essay into three stages, with both obligatory and optional moves. Querol and Madrunio (2020, p. 65) write, “in most of the argumentative essays, the three stages with the obligatory moves were followed although some new moves were also identified.” The three stages and moves at each stage are (Hyland, 1990, p. 69):

(1) **Thesis stage:** introduces the proposition to be argued Moves: (Gambit), (Information), Proposition, (Evaluation), (Marker)
(2) **Argument stage:** discusses grounds for thesis Moves: (Marker), (Restatement), Claim, Support
(3) **Conclusion stage:** synthesizes discussion and affirms the validity of the thesis Moves: (Marker), Consolidation, (Affirmation), (Close)

The moves in brackets show that these moves are optional instead of being obligatory to be found in an L2 argumentative essay. The rationale is that Hyland (1990)’s model offers detailed explanations of the structural units and corresponding functions (e.g., introduces the proposition to be argued) in an L2 argumentative essay, which can function as a backup in locating the identified extended metaphors in an L2 argumentative essay and then analyzing the intended rhetorical functions of extended metaphors. In line with the research on communicative functions of metaphors in academic texts or discourses (e.g., Goatly, 2011; Herrmann, 2013), the investigation on what rhetorical functions that extended metaphors can serve in different stages of an L2 argumentative essay, as well as in relation to each other, is also summarized by using Halliday and Matthiessen’s (2004) framework of three metafunctions of language: ideational, interpersonal and textual. The focus of this current investigation is on identifying extended metaphors in Chinese English learners’ argumentative texts, analyzing the communicative functions, and exploring learners’ thinking processes behind their production of extended metaphors during the writing processes. This article may hopefully contribute to the growing body of knowledge about learners’ metaphoric competence in L2 by recognizing L2 learners’ awareness and ability to create new and figurative meanings via extended metaphors, and to generate pedagogical implications in helping “students create their own, as opposed to text- or teacher-made, metaphors” (Darian, 2000, p. 184). While some researchers have used both text data and learner interviews to explore the influence of L1 and metaphoric thinking in L2 learners’ metaphor production processes (e.g., Xu and Tian, 2012; Hoang, 2015; Wang and Wang, 2019), none has shifted the focus for the use of extended metaphors in L2 writing and has had access to the writers talking about their thinking processes, or intentions, behind extended metaphor uses, so no possibility of eliciting their understandings and awareness of using extended metaphors during the writing processes. This gap was attempted to be filled.

Three research questions are addressed in this article:

(1) In what ways do Chinese learners of English use extended metaphors in their L2 argumentative essays?
(2) What are the communicative functions of extended metaphors when intertwined with the strategic moves and stages of an L2 argumentative essay?
(3) How do Chinese learners of English report their thinking processes behind extended metaphor uses during their writing processes?

## Materials and Methods

### Identifying Extended Metaphors and Establishing Systematic Metaphors

To answer the first two research questions, 72 argumentative writing samples were collected which were produced by 37 intermediate Chinese English majors in March and April 2018, on the abstract writing themes of *Spend and Save* and *Campus Love*. The second semester of each academic year in Chinese universities usually starts in March. In this semester, the learning objective of the writing module studied by the participants was argumentative writing. The participants also needed to practice argumentative writing as part of the preparation for their TEM-4 test (a national English language proficiency test for second-year English majors on the third Saturday of April every year in mainland China). This enabled a collection of authentic writing samples in a natural and principled way, without imposing additional work on both teachers and students. The linguistic metaphors were identified out by following the MIP (“metaphor identification procedure”) (Pragglejaz Group., 2007). The core principle of MIP is to compare the more abstract contextual meaning of a lexical unit with a more “basic” or concrete meaning in other contexts and look for a relation of comparison. Online versions of Macmillan Dictionary (Macmillan Education, London) and Oxford English Dictionary (Oxford English Press, University of Oxford) were consulted to establish the basic meaning and contextual meaning of each lexical unit and to minimize subjectivity in doing so. Following MIP, metaphor, metonymy, and simile were included as metaphorical when there were metaphor-related meanings. Then, the focus was turned to the identification of extended metaphors. As noted above, the extension of linguistic metaphors involves “at least two metaphorically used words belonging to different phrases describe the same target (topic) domain in terms of the same source (vehicle) domain (Semino, 2008, p. 25).

An example of extended metaphor is given in **Extract 1**, which is taken from one of the participants’ writing samples on the topic “The Reasons for College Students to Learn to Budget Their Money”:

***Extract 1** Once we want to waste money, the beasts of desire in our chests are awakened, they yell and stamp their feet, trying to control our mind.*

*(Deng, writing assignment submitted on 21/03/2018)*

**FIGURE 1:**
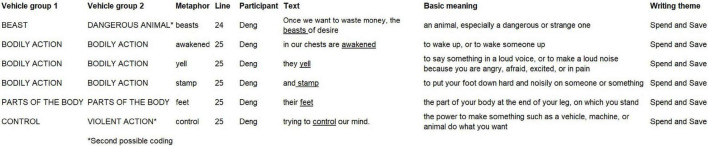
Possible groupings of metaphor vehicles in **Extract 1**.

The linguistic metaphors were underlined which were identified by following the MIP (Pragglejaz Group., 2007). The participant (Deng) used the clustering of connected linguistic metaphors and directly compared the desire to waste money as a horrible beast that can be awakened and cause a physical fight, violently threatening life. To avoid overgeneralization about writers’ conceptualization and intention in argumentative writing, Cameron and Maslen’s (2010) applied linguistic approach was followed to identify groupings that they term “systematic metaphors” by establishing “vehicle groupings” from collected linguistic metaphors in the discourse activity. The bottom-up approach of establishing systematic metaphors from extended metaphors is not the same as the generalization of conceptual metaphors in the Conceptual Metaphor Theory (CMT). The latter has been problematic for metaphor research focusing on naturally occurring data in context because of its use of invented linguistic evidence and its top-down approach of apparently preselecting conceptual metaphors then tracking for evidence of their realizations at the linguistic level (Cameron, 2010; Deignan, 2010). From finding systematic metaphors from semantically connected metaphor vehicles, researchers aim to “draw inferences about their [participants’] thoughts and feelings, their [participants’] conceptualizations and communicative intentions, from the language they [participants] used then” (Maslen, 2017, p. 89). The “systematic metaphors” termed by Cameron et al. (2010) resemble the conceptual metaphors suggested by the CMT, “but they should not be seen as equivalent” (Deignan et al., 2013, p. 9). In this article, systematic metaphors were then established by following Cameron et al. (2010) practice of grouping metaphor vehicles by using the Excel software (Microsoft, United States). The semantics of the basic meaning of the metaphor vehicles were used as the starting point to generalize grouping labels (see Figure 1).

In Figure 1, “linguistic metaphors were gathered together in a list and then were grouped and organized according to the basic meanings of the vehicle terms” (Cameron, 2010, p. 12). The grouping labeled *BODILY ACTION* included the linguistic metaphors “awakened,” “yell” and “stamp” in **Extract 1**. The grouping labeled *BEAST* was first generalized from the explicit metaphorical expression “the beasts of desire” and then was further grouped into *DANGEROUS ANIMAL* in terms of the basic meaning of “beast”: “an animal, especially a dangerous or strange one,” according to online Macmillan Dictionary. So, at the very beginning, the “labels for groupings were often taken from the actual words that appear in the written data” (Cameron et al., 2010, p. 119) and the words that appear in the basic meanings of metaphor vehicles. This process “contracts with Conceptual Metaphor Theory which aims to generalize labels as much as possible in order to posit universals in human conceptualizing” (Cameron et al., 2010, p. 119). The second possible grouping labeled *VIOLENT ACTION* was one of the two subdivisions of the *PHYSICAL ACTION* grouping. The *PHYSICAL ACTION* metaphor vehicles can be further divided into *PHYSICAL ACTION* and *VIOLENT ACTION* in terms of “those actions which are neutral and those which express an element of violence” (Cameron et al., 2010, p. 123). Based on the immediate text context in **Extract 1**, and the basic meanings of collected metaphor vehicles — “beasts,” “yell” and “stamp,” the grouping labeled *CONTROL* was further generalized as *VIOLENT ACTION*. The grouping *PARTS OF THE BODY* was quickly built by referring to both the basic meaning of “feet” and the grouping *PARTS OF THE BODY* in Cameron and Maslen’s (2010) work. “A systematic metaphor is a set of linguistic metaphors in which connected vehicle words are used metaphorically about a particular topic” (Cameron et al., 2010, p. 127). It was easy to find the topics based on the immediate writing contexts and the writing themes in collected text data. For example, in the *DANGEROUS ANIMAL* grouping in Figure 1, a subset of metaphor vehicles that were used to talk about the desire of wasting money were connected and grouped together as the systematic metaphor: *DESIRE OF WASTING MONEY IS A DANGEROUS ANIMAL WITH VIOLENT BODILY ACTION.*

The bottom-up procedure of finding systematic metaphors from vehicle groupings generalized in extended stretches of written texts focuses on what the communicative intentions or goals are when the participants used extended metaphors at some strategic moves and stages in L2 argumentative texts (Deignan et al., 2013; Deignan, 2017). Systematic metaphors established from the extended metaphors identified in writing samples serve both as evidence for ideas, attitudes, and values which may not be directly expressed in the texts, and as a starting point for the further exploration of functions of metaphor clusters (Cameron et al., 2010, p. 116). As mentioned above, Hyland’s (1990) model of describing the rhetorical structure of an ESL argumentative essay and Halliday and Matthiessen’s (2004) framework of three metafunctions of language: ideational, interpersonal, and textual, are the theoretical guide. In the example analysis of **Extract 1**, the textual function of extended metaphors, such as “creating internal coherence” (Koller, 2003, p. 120), can be realized by the connected metaphor vehicles that can be summarized by the metaphorical idea—“beasts of desire” at the argument stage of the writing sample (Cameron and Low, 2004). The new representations of the desire of wasting money in terms of a dangerous animal are evidence of the ideational functions of extended metaphors (Corts and Pollio, 1999; Goatly, 2011; Kathpalia and Carmel, 2011) in the move of making a claim. The systematic metaphor *DESIRE OF WASTING MONEY IS A DANGEROUS ANIMAL WITH VIOLENT BODILY ACTION* not only contributes to building a coherent argument (textual function) but also a persuasive one (interpersonal function) at the argument stage of Deng’s writing sample. The *BEAST* metaphor is used to describe crime in Thibodeau and Boroditsky’s (2011) research, and the participant creatively extends the *BEAST* metaphor when arguing about the reasons and importance of saving money. In **Extract 1**, the systematic metaphor highlights the negative elements and deemphasize the positive ones contained in the topic domain *DESIRE OF WASTING MONEY* (Thibodeau, 2017), to affect readers’ concerns and beliefs and to persuade them to take specific actions (interpersonal function) (Hyland, 1990; Cameron and Maslen, 2010; Paquot, 2010; Goatly, 2011; Littlemore et al., 2014; Yang et al., 2014; Thibodeau, 2017).

### Stimulated Recall Interviews

“Stimulated recall methodology can be viewed as a subset of introspective research methods which help the researchers to accesses, examine and understand participant’s reflections on mental processes” (Gazdag et al., 2016, p. 119; Fox-Turnbull, 2011, p. 205). Prior research has demonstrated that “stimulated recall methodology can be used to prompt participants to recall thoughts they had while performing a task or participating in an event” (Gass and Mackey, 2000, p. 13; Mackey and Gass, 2005; Henderson et al., 2010; Fox-Turnbull, 2011; Ryan and Gass, 2012; Gazdag et al., 2016; Gass and Mackey, 2017). The application of stimulated recall methodology to L2 research has been extended from investigating classroom practices and interactions like videotaped lectures or discussions to exploring participants’ mental processes in events like reading and writing (Gass and Mackey, 2017). Hoang (2015) used keystroke data generated by the Input-Log program together with the stimulated recall interviews to explore how Vietnam learners of English explained their metaphors used in their in-class compositions based on an elicitation writing task prepared by the researcher. Hoang (2015) used the stimulated recall interviews, with well-prepared interview protocol and instructions for both researcher and the students, to reveal the underlying factors that may directly link to the development of metaphorical units in students’ topic-based writing, by transcribing and categorizing the participants’ comments. The three outstanding categories in her participants’ comments on metaphor uses were “the use of images, background knowledge, and novel metaphors” (Hoang, 2015, pp. 97–98). As Wang and Cheng (2016) suggest, “probing factors behind learners’ metaphoric creativity can thus enrich teachers’ knowledge of how to develop learners’ ability to use L2 metaphorically, preparing them to participate in actual social communication” (Wang and Cheng, 2016, p. 205). Extended metaphors in texts or discourses are often linked with novel or creative metaphorical ideas and intended communicative purposes (Denroche, 2018). By now, the investigation of extended metaphors and metaphoric creativity in Chinese English learners’ L2 argumentative writing, and the examination of possible factors underlying L2 learners’ metaphor use in writing still seems to be an under-researched area. To answer the third research question and to contribute knowledge of learners’ understanding of their writing process in terms of extended metaphor use, stimulated recall interviews were conducted. Each individual interview (around 30 min) was conducted within 2 days of the submission of the related writing sample to maximize the recall accuracy. The stimulated recall methodology was piloted with five of targeted participants at the very beginning of the data collection procedure. The aim of this was to decrease the amount of unnecessary information in the interviews and help the participants to focus on the recalling process. The audio-recorded interview data was manually transcribed and then translated from Chinese into English following strict conventions (Richards, 2003, pp. 80–81; Watanabe and Swain, 2007, p. 140; Bailey, 2008, p. 131). Supporting evidence of possible intended functions of learners’ extended metaphor uses is also hoping to be found in the stimulated recall interview data. Participants were asked two key interview questions: (1) When writing words or phrases like this, what were you thinking about or how did you perceive it? (2) Why did you use this/these particular word/words or phrases, what were you thinking about then?

An interview extract from the interview with Deng, the author of **Extract 1**, is given below:

***Researcher:** Yeah, you used “Once we want to waste money,” you wrote, “the beasts of desire in our chest are awakened,” so why you expressed like this at that particular time?*

***Deng:** I wanted to be more vivid. I just wanted to stress again that our desire, the importance of controlling that kind of desire. Because what I wanted to say was that desire was like a dreadful monster. If it were awakened, you would be out of control.*

From Deng’s self-reports, her conscious reflection on her desire to make the writing more vivid and her metaphoric thinking of “desire” as “a dreadful monster” at the time of writing have been clearly and confidently verbalized. By emphasizing the negative effect of not controlling desire well, Deng’s metaphorical extension of the *BEAST* metaphor could support her viewpoint and argument and reinforce the persuasive nature of the argumentative essay. The stimulated recall method has been able to generate interesting insights when efforts have been made to ensure that the accurate recall has been taken place, which may support the function analysis of the systematic metaphors established from the extended metaphors involving single metaphorical ideas, e.g., the pervasive power of *DESIRE OF WASTING MONEY IS A DANGEROUS ANIMAL WITH VIOLENT ACTION* metaphor established from Deng’s writing sample. The study hoped to draw teachers’ attention to learners’ conscious uses of extended metaphors in argumentative texts and to enrich teachers’ knowledge of metaphors in developing learners’ metaphoric competence in L2.

## Findings

### Extended Metaphors and Communicative Functions

In total, I identified 11 single extended stretches from the written texts produced by 9 writers, including **Extract 1** illustrated above for demonstration purposes, from which 11 systematic metaphors were established (see Table 1). The underlined metaphor vehicles are linguistic metaphors extended in single metaphorical ideas.

**TABLE 1:** Examples of extended metaphors and systematic metaphors.

Systematic metaphors	Extended stretches	Participants	Topics
*DESIRE OF WASTING MONEY IS A DANGEROUS ANIMAL WITH VIOLENT ACTION*	Once we want to waste money, the beasts of desire in our chests are awakened, they yell and stamp their feet, trying to control our mind.	Deng	Spend and save
*MONEY WITHOUT CONTROL IS A WILD ANIMAL*	Without any goals, your money will run wild and go all over the place. You will even find that you buy nothing useful which causes a lot of money. Take control of your money and stick to your goals.	Shi	Spend and save
*MONEY IS A PERSON*	“Money is good servant but a bad master.” We cannot be controlled by money so that we will not be a pathetic slave of money.	Li N.	Spend and save
*SPENDING IS A VEHICLE*	There is a common view in China saying that the three carriages of the economy are consumption, export, and investment. […] we should pay a lot attention to consumption and rationally spend more so that our economy can have a sustainable and powerful driving force.	Wang	Spend and save
*SAVING MONEY IS RESERVING WEAPON*	If life is compared to a war, saving money is like to storage [store] bullets.	Zhang	Spend and save
*LOVE IS PHYSICAL FORCE DRIVING VEHICLES/MACHINES*	Basically, love is the invisible power. It has the driving force which can encourage people to achieve some goals. […] love is like the petrol to a car, the battery to a player.	Li Y.	Campus love
*NEGATIVE EMOTION CAUSED BY BREAKUP IS A BOMB*	Campus love will increase the psychological burden. When they experience a breakup, they will feel desperate and depressed for a long time. Their feelings will be a ticking time bomb which will lead to terrible consequences when it blows up.	Liu	Campus love
*LOVE IS ILLNESS*	Love can be a good medicine or a poison. […] we all know that romance can go sour, and a failed love experience can be stressful and painful.	Lou	Campus love
*LOVE IS FIRE*	Love is like a fire, warm and bright but easy to burn.	Lou	Campus love
*LOVE IS FOOD BEARING PLEASANT FEELINGS*	Pursuing romantic love is the instinct of human, just like hungry people find [finding] food. […] the romantic love, just like desert [dessert] can make the hearts warm and happy.	Guo	Campus love
*HUMAN BEINGS ARE PLANTS AND ANIMALS*	Only when flowers bloom will the bees come to gather honey.	Deng	Campus love

During the process of establishing systematic metaphors, there were situations where one metaphor vehicle could be grouped into different vehicle groupings. Collaborative decisions were made to group one metaphor vehicle into one most appropriate vehicle grouping for the ease of categorization (Cameron et al., 2010). The metaphor vehicles “petrol” and “battery” in **Extract 2**, on the topic “More Than Love,” for example:

***Extract 2** Basically, love is the invisible power. It has the driving force which can encourage people to achieve some goals. […] love is like the petrol to a car, the battery to a player.*

*(Li Y., writing assignment submitted on 28/03/2018)*

first were grouped as *ENERGY* because of the words like “fuel” and “electricity” in the basic meanings of metaphor vehicles, based on the online Macmillan Dictionary. After regular discussion with co-rater who has a professional background in metaphor research, the group was then recorded and broadened to *VEHICLE* by including metaphorically used words—“car” and “player” representing vehicles and machines in the physical world. As noted above, the metaphor vehicle “control” in **Extract 1** was grouped as *VIOLENT ACTION* by following Cameron and Maslen’s (2010) two subdivisions of the *PHYSICAL ACTION* grouping. The rationale is that the context of the *BEAST* metaphor may convey a sense of violence. Borderline cases about the metaphor vehicle “control,” which can be grouped into *VIOLENT ACTION* or *PHYSICAL ACTION* depending on writing contexts were agreed upon after discussion. The bilingual background of the co-rater and the author, and their familiarity with Chinese intermediate English learners’ argumentative writing were helpful in capturing accurate generalizations of the metaphor vehicles and the corresponding topics to which participants had written. The trustworthiness of vehicle groupings can be maximized by “keeping with the ‘principled flexibility’ that has informed the process throughout” (Cameron et al., 2010, p. 126). By regular discussions with co-rater throughout the metaphor identification and metaphor analysis processes, the systematic metaphor proposed from the extended metaphorical stretch in **Extract 2** is: *LOVE IS PHYSICAL FORCE DRIVING VEHICLES/MACHINES*.

**FIGURE 2:**
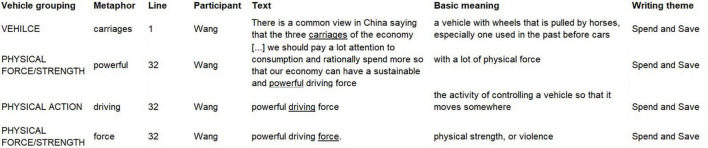
Extended metaphor contributing to the *VEHICLE* systematic metaphor.

**FIGURE 3:**
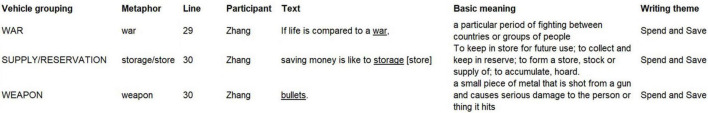
Extended metaphor contributing to the *SAVING MONEY IS RESERVING WEAPONS* systematic metaphor.

**FIGURE 4:**
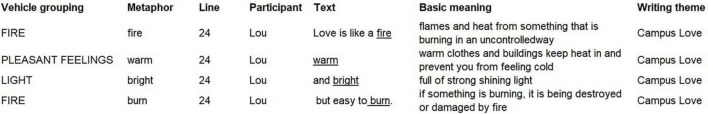
Extended metaphor contributing to the *FIRE* systematic metaphor.

**FIGURE 5:**
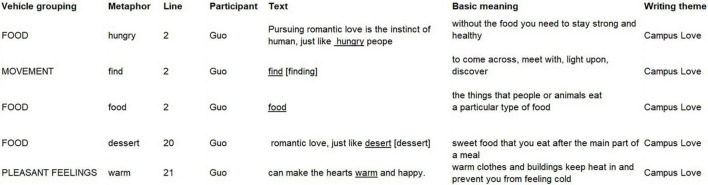
Extended metaphor contributing to the *FOOD* systematic metaphor.

Like the functional analysis of the *BEAST* metaphor noted above, the systematic metaphor *LOVE IS PHYSICAL FORCE DRIVING VEHICLES AND MACHINES* could contribute to building a coherent argument (textual function) but also a persuasive one (interpersonal function) at the argument stage of the writing text. The student creatively used novel metaphors to increase comprehensibility and to highlight the positive role of campus love as *PHYSICAL FORCE/STRENGTH* (ideational function), which were possible attempts made to persuade the readership to accept the writer’s viewpoint (interpersonal function). More examples of functional analysis of the identified extended metaphors listed in Table 1 are given (see Figures 2–5).

In Figure 2, spending (topic domain) is described as a vehicle (vehicle domain) *via* four metaphorically used words in the gambit move of the thesis stage and the closing move of the conclusion stage in Wang’s writing text. This extension coincides with the gambit move in an argumentative essay where the writer’s purpose is to “capture the readers’ attention, rather than inform” (Hyland, 1990, p. 70) by way of dramatic illustration at the very beginning of the writing text. These linguistic metaphors are related to a vehicle that can move forward. A systematic metaphor *SPENDING IS A VEHICLE* can be formulated, offering the topic of spending a new representation and the text’s internal coherence. *SPENDING IS A VEHICLE* used when introducing viewpoints on the positive side of spending can convey a writer’s positive attitude toward spending and then construct the evaluative function of metaphor (Goatly, 2011). Attempts contained in this systematic metaphor, such as dramatic illustration, coherence construction, and evaluation, can be related to the three dimensions of functions of language: ideational, interpersonal, and textual.

In Figure 3, linguistic metaphors “war,” “storage [store],” and “bullets” are used creatively to talk about the topic of money (topic domain) in terms of weapon (vehicle domain), at the conclusion stage of Zhang’s writing text. The words in square brackets are some grammatical mistakes corrected by the researcher with participants’ agreement. Bullets can be supplied or reserved and used for war. A creative systematic metaphor, therefore, is formulated: *SAVING MONEY IS RESERVING WEAPONS*. The ideational, interpersonal, and textual function of this systematic metaphor is intertwined with the rhetorical goal of the conclusion stage, which is to summarize the argument section persuasively, to provide a prospective focus for discussion, and to achieve vivid consolidation (Hyland, 1990; Querol and Madrunio, 2020). It seems that the conventional metaphoric idea *LIFE IS WAR* is compatible with the creative systematic metaphor *SAVING MONEY IS RESERVING WEAPONS* concerning the connection between war and weapons.

In Figure 4, the conventional systematic metaphor *LOVE IS FIRE* built from the metaphorical extension at the conclusion stage in Lou’s writing text also can help to realize the persuasive power in the writing text in a coherent and dramatic way.

In Figure 5, love (topic domain) is described as food (vehicle domain) *via* three different linguistic metaphors (“hungry,” “food,” “dessert”) within the same text. The connection among these linguistic metaphors could be reflected in the metaphor flag “just like.” Here, this subset of metaphor vehicles, consisting of extended metaphor, is used to talk about love, and can be expressed through the systematic metaphor: *LOVE IS FOOD BEARING PLEASANT FEELINGS*. The conventional concepts *LOVE IS FOOD* or *LOVE IS SWEET FOOD* are lexicalized by the creative and possibly deliberate comparisons between “pursuing love” and “finding food,” and between “love” and “dessert.” The linguistic metaphors “hungry” and “food” at the thesis stage give a focus to Guo’s proposition, and “dessert,” “warm,” and “happy” at the argument stage reinforce his claim (Hyland, 1990). So, clusters that include extended metaphors at different moves and stages could give internal coherence to an argumentative text, which is the textual function of extended metaphors. Guo’s creative use of these similes highlights the positive side of love, which conveys evaluative and persuasive power, i.e., interpersonal function.

The bottom-up analysis of systematic metaphors shows extended metaphors are often found to build coherent and persuasive arguments in learners’ written texts. Among the 11 instances of systematic metaphors, seven occurred at the argument stage, one at both thesis stage and argument stage, one at both thesis stage and conclusion stage, and two at the conclusion stage. The *SPENDING IS A VEHICLE* metaphor is found at both the thesis and conclusion stage in Wang’s writing sample, contributing to the textual structuring function and ideational function simultaneously. The two systematic metaphors built at the conclusion stage of the writing samples can be sensed as deliberate and creative because of similes. The systematic metaphor *LOVE IS FIRE* in the close move in Lou’s text reinforces the evaluative and persuasive power. The systematic metaphor *SAVING MONEY IS RESERVING WEAPON* at the conclusion stage in Zhang’s text conveys the writer’s strong emotions toward the importance of saving money, which might encourage a change of perspective. The *DESIRE OF WASTING MONEY IS A DANGEROUS ANIMAL WITH VIOLENT ACTION* metaphor and *LOVE IS PHYSICAL FORCE DRIVING VEHICLES/MACHINES* metaphor established from the argument stages also contribute to the construction of coherent and persuasive arguments. The function analysis focusing on extension and systematicity generates insights on how Chinese learners of English use extended metaphors at strategic points in their argumentative essays, and for what communicative purposes. More supporting evidence on learners’ intentions and communicative purposes are obtained from the follow-up stimulated recall interviews.

### Learners’ Thought Reports in Stimulated Recall Comments

Not all extended metaphors identified are able to be asked in the stimulated recall interviews because of the time limitation and ethical considerations. Due to the difficulty of finding a time to interview students within 2 days of the writing tasks, four extended stretches listed in Table 1 (including **Extract 1** explained above for demonstration purpose), written by four different participants, were able to be asked in the stimulated recall interviews. The four participants were interviewed individually in a face-to-face manner during their free time. Two interviews were on *Spend and Save* and two on *Campus Love*. All interviews were voluntary and did not cause extra workload to both teachers and students involved in this present investigation. Table 2 presents participants’ thought reports cited in their recall comments.

**TABLE 2:** Extended stretches and corresponding recall comments.

Extended stretches	Thought reports cited in recall comments
Once we want to waste money, the beasts of desire in our chests are awakened, they yell and stamp their feet, trying to control our mind.	**Deng:** “I wanted to be more vivid. I just wanted to stress again that our desire, the importance of controlling that kind of desire. Because what I wanted to say was that desire was like a dreadful monster. If it were awakened, you would be out of control”.
There is a common view in China saying that the three carriages of the economy are consumption, export, and investment. […] we should pay a lot attention to consumption and rationally spend more so that our economy can have a sustainable and powerful driving force.	**Wang:** “What I was thinking at that moment is that, first, the topic is economy and spending, and then I came up with the same Chinese expression that I learned in senior high school so I translate the ‘马车’ into ‘carriages.’ The three carriages are equal to the driving power of economic development. […] I wanted to echo the earlier expression “three carriages,” so I wrote ‘powerful driving force,’ which means the drive that can lead to economic development”.
Basically, love is the invisible power. It has the driving force which can encourage people to achieve some goals. […] love is like the petrol to a car, the battery to a player.	**Li Y.:** “It was, when I was using English to express myself, I worried that the readership might not understand my intended meaning. Maybe there was some of my own subjective understanding in it. I just wanted to mean that love is a strength that can move things forward, just like the function of petrol to a car and the batteries in a player. The strength was dominating because it could make you alive and give you energy, and make you operate and work. This is what I was thinking”.
Pursuing romantic love is the instinct of human, just like hungry people find food. […] the romantic love, just like desert [dessert], can make the hearts warm and happy.	**Guo:** “Here I just want to make it clear that love is positive. It is normal and common, with no negative side. […] Love is sweet. When thinking about sweety, it is easy for me to link with desserts”.

At this stage, the focus was on what the participants said about their choice of metaphorical expressions that were identified as extended metaphors. The participants were not told whether a stretch of written texts had been classified as a metaphorical extension before or during the interview processes. Each student was interviewed no more than once. The opening coding approach, on a line-by-line basis (Richards, 2003), enabled to constantly comparing the similarities and differences among learners’ comments on their metaphor use at the time of writing when coding recall data. Here, “a code ascribes meaning to the coded text” (Jamieson, 2016, p. 8). So, the explanations and thought reports that are similar at the conceptual level could be grouped into themes or categories by breaking down the interview data for the analytical purpose (Corbin and Strauss, 1990; Chapman et al., 2015). As demonstrated above, it was observed that learners were willing to discuss their language uses with me. Learners also reported on their conscious or deliberate metaphor uses at the time of writing. Table 3 illustrates the four categories of reasons that were identified for learners’ extended metaphor uses.

**TABLE 3:** Grouping codes into themes.

Coded recall comments	Codes (C#)	Themes (T#)
I wanted to be more vivid. I just wanted to stress again that our desire, the importance of controlling that kind of desire. Because what I wanted to say was that desire was like a dreadful monster.	C1: Compare one abstract concept to a more concrete one to achieve vividness C2: Compare one abstract concept to a more concrete one by looking for similarities C3: Desire to persuade through metaphorical constructions	T1: Metaphoric thinking (Littlemore and Low, 2006a) T2: Communicative functions of metaphor in academic writing (Goatly, 2011; Herrmann, 2013)
What I was thinking at that moment is that, first, the topic is economy and spending, and then I came up with the same Chinese expression that I learned in senior high school so I translate the ‘马车’ into ‘carriages.’ The three carriages are equal to the driving power of economic development […]	C4: Use the first language as a base for understanding or producing the second language (O’Malley and Chamot, 1990, p. 120)	T3: L1 Influence (Ellis, 1999; Nacey, 2013)
I wanted to echo the earlier expression “three carriages,” so I wrote “powerful driving force,” which means the drive that can lead to economic development.	C5: Desire to make the writing coherent	T2: Communicative functions of metaphor in academic writing (Goatly, 2011; Herrmann, 2013)
It was, when I was using English to express myself, I worried that the readership might not understand my intended meaning. Maybe there was some of my own subjective understanding in it.	C6: Struggling to express meaning	T4: Limited L2 knowledge and desire for a better writing performance in L2 (Hinkel, 2002; MacArthur, 2010)
I just wanted to mean that love is a strength that can move things forward, just like the function of petrol to a car and the batteries in a player. The strength was dominating because it could make you alive and give you energy, and make you operate and work.	C2: Compare one abstract concept to a more concrete one by looking for similarities C3: Desire to persuade through metaphorical constructions	T1: Metaphoric thinking (Littlemore and Low, 2006a) T2: Communicative functions of metaphor in academic writing (Goatly, 2011; Herrmann, 2013)
Here I just want to make it clear that love is positive. It is normal and common, with no negative side. […] Love is sweet. When thinking about sweety, it is easy for me to link with desserts.	C2: Compare one abstract concept to a more concrete one by looking for similarities C3: Desire to persuade through metaphorical constructions	T1: Metaphoric thinking (Littlemore and Low, 2006a) T2: Communicative functions of metaphor in academic writing (Goatly, 2011; Herrmann, 2013)

Based on the interview data, the four reasons explaining when and why learners’ produce extended metaphors are: (1) learners’ metaphoric thinking; (2) communicative functions of metaphor in academic writing (ideational, interpersonal, and textual); (3) L1 influence; (4) learners’ limited L2 knowledge and desire for a better writing performance in L2. It seemed that more than one reason was cited concerning each of the extended metaphor use identified and asked in this present investigation, which shows participants’ ability, or efforts made, to think, write, and persuade metaphorically and creatively in English, i.e., learners’ metaphoric competence in L2. In the following section, the findings obtained from learners’ writing samples and stimulated recall interviews were discussed.

## Discussion of Findings

Findings obtained from the written texts data indicate that extended metaphors can be found at different strategic points in Chinese English learners’ argumentative essays. Functions of extended metaphors are analyzed by taking the strategic moves and stages of an L2 argumentative essay (Hyland, 1990) into consideration. Like what Koller (2003) has found by analyzing metaphor clusters in magazine texts on marketing, it has been found that extended metaphors in the mid-texts, i.e., the argument stages, often serve the interpersonal function, such as developing persuasive arguments. The extended metaphors at the thesis stages, and at the concluding stages of participants’ argumentative texts, often occur on smaller scales compared to those in the middle parts. The bottom-up analysis of systematic metaphors built from extended metaphors indicates that communicative functions of extended metaphors at the beginning of written texts often coincide with the rhetorical aims of the thesis stage, such as achieving a dramatic illustration and attracting the readership’s attention, which are the ideational and interpersonal function. Extended metaphors at the end of texts can help learners to reinforce the proposition by “providing a prospective focus and widening the context” (Hyland, 1990, p. 74). Learners can “drive the point home to the readership” (Koller, 2003, p. 120) and achieve persuasive power.

Some examples of extended metaphors which appear to be deliberate were also observed. More than one systematic and metaphoric idea can be found within the same text. For instance, in Lou’s writing sample, the systematic metaphor *LOVE IS ILLNESS* can be established from the extended metaphors used at the argument stage for presenting and supporting standpoints. The systematic metaphor *LOVE IS FIRE* can be built from the conclusion stage to reinforce the central viewpoint and widen the context for evaluation and persuasion. This indicates that Lou could deploy and develop different vehicle terms and metaphoric ideas to talk about the topic domain at different stages of the text, with stronger emotions and persuasive power. The *LOVE IS FOOD* metaphor across the thesis and argument stages of Guo’s writing sample could also show a degree of learners’ conceptual fluency and metaphoric competence in L2 English (Danesi, 1992). Different systematic metaphors built from different extended texts show learners’ ability to facilitate change in perspectives on part of the readership, by directing the readership’s attention and understanding to a different area of experience (Deignan et al., 2013). The function analysis of extended metaphors, by establishing systematic metaphors, provides more evidence about learners’ metaphoric competence in L2, at both conceptual and linguistic levels of metaphor (Littlemore, 2010; Nacey, 2013).

The stimulated recall interviews enabled to talk to learners and know more about their intentions and purposes in terms of their choice of some metaphorical extensions. Learners’ thought reports cited in their recall comments suggest that they are confident about their word choices during their writing processes, no matter whether the words and expressions are deliberately used to be metaphorical or not. Learners are able to report clearly about the efforts they have made to express their meanings during the writing, such as directly applying the metaphorical comparison from L1 to L2 and consciously thinking metaphorically in L1. Learners also report their desire for vividness, better comprehensibility, and persuasive power, concerning some extended metaphor uses, which supports the function analysis of extended metaphors and learners’ metaphoric competence in L2. The stimulated recall methodology has its limitations, but it is believed to be enough for this present investigation to ask learners in a face-to-face manner to know more about their thinking processes behind their use of extended metaphors in L2 argumentative texts. Useful pedagogical implications can be obtained. For instance, there are situations where learners may consciously decide to use extended metaphors to persuade through metaphorical constructions. Now that evidence has shown that both conventional and creative extended metaphors are inevitable for learners to meet various communicative needs in writing, it is necessary for teachers to realize this, recognize this as not an arbitrary phenomenon but a way of thinking and communication, and offer corresponding feedback in developing learners’ metaphoric and communicative competence in L2. The interesting insights obtained from the analysis of stimulated recall interview data can offset the limitations of the stimulated recall methodology.

Learners’ thought reports cited in some recall comments may also provide supporting evidence to the possible presence of certain metaphorical ideas in L1 or L2, such as “desire was like a dreadful monster” (Deng) and “love is a strength that can move things forward” (Li Y.), in the writers’ minds when they wrote extended metaphors. This may contribute to, as Littlemore (2009) suggests, the implications of CMT in second language teaching and learning. L1 influence on L2 metaphor production is not “simply lexical interference from the L1, or as the result of a knowledge gap in the use of L2 idiomatic language” (Danesi, 2016), but also may be the result of conceptual transfers from L1 to L2 (Nacey, 2013).

## Conclusion

This present investigation is a relatively small-scale study. The collected text data and interview data may not represent all Chinese university students’ use, function, and understanding of extended metaphors in L2 argumentative writing. However, limitations like these are less important compared to the findings and insights gained from the textual analysis and interview analysis. The findings from this present investigation show that Chinese learners of English have been able to refer to some metaphorical concepts in their L1 to produce conventional and creative extended metaphors in L2 argumentative texts for achieving various communicative purposes, such as the desire for vividness, for more comprehensible meaning, coherence, and for supporting viewpoints and persuading. Findings from the interview data also show that learners may develop metaphorical extensions deliberately, by consciously thinking metaphor in L1 and writing creative direct metaphors under certain communication pressure. But participants’ ability to write metaphorically in their targeted language, and sometimes to report metaphorically about their writing processes, are still not recognized as a crucial ability to be developed in their L2 classrooms. Littlemore and Low write, “control over metaphor is one of the essential tools for empowering learners to cope successfully with native speakers” (Littlemore and Low, 2006b, p. 22). It is reasonable to constantly draw both teachers’, learners’, and policy makers’ attention to the exposure of metaphor knowledge in L2 classrooms at the tertiary level (Shirazi and Talebinezhad, 2013), and pay more attention to learners’ metaphor production in L2 (Hoang, 2015). The reinforcement of metaphor awareness, metaphoric/creative thinking, and cross-cultural awareness is essential in developing Chinese English learners’ metaphoric competence and overall communicative competence in L2.

## Data Availability Statement

The original contributions presented in the study are included in the article/supplementary material, further inquiries can be directed to the corresponding author.

## Ethics Statement

The studies involving human participants were reviewed and approved by the ESSL, Environment and LUBS Faculty Research Ethics Committee, University of Leeds (AREA 16-160). The patients/participants provided their written informed consent to participate in this study.

## Author Contributions

QL contributed to the conception and design of the study, conducted data collection, performed the analysis and interpretation of both text and interview data, wrote the first draft, and made the revisions and approved the publication of this article.

## Conflict of Interest

The author declares that the research was conducted in the absence of any commercial or financial relationships that could be construed as a potential conflict of interest.

## Publisher’s Note

All claims expressed in this article are solely those of the authors and do not necessarily represent those of their affiliated organizations, or those of the publisher, the editors and the reviewers. Any product that may be evaluated in this article, or claim that may be made by its manufacturer, is not guaranteed or endorsed by the publisher.

## Footnotes

1 In this article, “systematic metaphors are written in *SMALL ITALIC CAPITALS* (e.g., *LANGUAGE IS A PERSON*) to distinguish them from conceptual metaphors (e.g., LOVE IS A JOURNEY), which are written in SMALL CAPITALS” (Cameron et al., 2010, p. 117).
